# Dietitians Australia position statement on healthy and sustainable diets

**DOI:** 10.1111/1747-0080.12726

**Published:** 2022-03-01

**Authors:** Liza Barbour, Ellyn Bicknell, Julie Brimblecombe, Stefanie Carino, Molly Fairweather, Mark Lawrence, Juliet Slattery, Julie Woods, Elizabeth World

**Affiliations:** ^1^ Department of Nutrition, Dietetics & Food Monash University Notting Hill Victoria Australia; ^2^ University of Melbourne Parkville Victoria Australia; ^3^ Deakin University Institute for Physical Activity and Nutrition (IPAN), School of Exercise and Nutrition Sciences Geelong Australia; ^4^ Te Atiawa Manawhenua ki te Tau Ihu Trust Waikawa New Zealand; ^5^ Dietitians Australia Deakin Australian Capital Territory Australia

**Keywords:** diet, environmental sustainability, planetary health, public health nutrition

## Abstract

It is the position of Dietitians Australia that to promote human and planetary health, a food system transformation is needed that enables the population to adopt healthy and sustainable diet‐related practices. A healthy and sustainable diet must (i) be nutritionally adequate, healthy and safe, (ii) have low environmental impact and be protective of natural resources and biodiversity, (iii) be culturally acceptable and (iv) be accessible, economically fair and affordable. Dietitians Australia acknowledges that it is critical to prioritise Indigenous knowledges in consultation, policy‐making and implementation processes to achieve these recommendations. In facilitating the uptake of healthy and sustainable diets, dietitians are contributing to the transformation of our current food system that is urgently required to nourish present and future generations within planetary boundaries. In developing this position statement, opportunities for future research have been identified including those to advance the professions' capacity to improve environmental sustainability outcomes across all areas of practice. To achieve a population‐level shift towards this diet, Dietitians Australia recommends: (i) the development of a National Food and Nutrition Strategy which honours Indigenous knowledges on food systems, (ii) the integration of sustainability principles in Australia's dietary guidelines, (iii) the reorientation of our food environment to prioritise access to healthy and sustainable foods, and (iv) investment in capacity building activities to equip the current and future nutrition and dietetics workforce.

## 1 BACKGROUND

Indigenous peoples of the world have managed sustainable food systems for millennia, providing food, livelihoods and well‐being to humankind.^1^ Indigenous people's food systems are founded on values of reciprocity and respect for the whole ecosystem, whereby humans are interconnected with the natural environment. The way food is produced and consumed has changed drastically over recent decades and disregards Indigenous knowledge of human–ecology interaction and its balance.^2^, ^3^ As we attempt to mitigate the effects of climate change and an increasing prevalence of diet‐related disease, Indigenous peoples' knowledges of sustainable food systems can provide insights, lessons and evidence.^1^, ^4^ It is believed that ‘the world cannot feed itself sustainably without listening to Indigenous peoples’.^1^ ^,p.10^ However, many would argue that to promote human and planetary health, the global community must go beyond listening and demonstrate a deep respect for Indigenous peoples as the custodians and expert stewards of the land, waterways and our finely balanced ecosystem.

The relationship between our climate and our food system is bidirectional. On one side, climate change is affecting our planet's ability to produce food under extreme weather conditions, diminishing natural resources, ocean acidification and rising sea levels.^5^, ^6^ On the other side, our food system disrupts natural ecosystems by creating more greenhouse gas emissions than any other single contributor, causing land degradation, depleting water stores and driving biodiversity loss.^7^, ^8^ While food systems have the potential to promote human health, environmental sustainability and equity, they are currently threatening all three.^2^ Poor quality diets remain the leading preventable risk factor for chronic disease, particularly amongst lower socio‐economic groups where inequities in access to resources prevail.^9^, ^10^ Despite evidence that global food production has kept pace with population growth in terms of dietary energy requirements, over 820 million people have insufficient food.^2^ The current food system is failing on both counts.

In Australia, the way our food is produced, manufactured, distributed and consumed is contributing to climate change and malnutrition in all its forms.^10^, ^11^ Our agricultural sector is responsible for 16% of Australia's greenhouse gas emissions as well as biodiversity loss, water consumption and unsustainable land management practices.^12^ Our dietary consumption patterns yield the highest per capita greenhouse gas emissions of all the G20 countries.^13^ If the global population were to adopt Australian consumption patterns, by 2050 the natural resources of over six and a half Earths would be required to support food production.^13^ This is only worsened by the fact that foods which are energy‐dense and nutrient‐poor account for 27% of diet‐related emissions.^14^, ^15^, ^16^ These discretionary products (foods high in fat, sugar and/or salt) contribute 38% of the energy purchased from food and drinks from supermarkets in Australia.^17^ Our food system is also a key contributor to chronic disease risk. Australia and New Zealand have the highest rates of childhood overweight at 16.9%, compared to a global average of 5.7%, and the highest rates of adult obesity at 30.7%, compared to a global average of 13.2%.^18^ Despite being considered ‘the lucky country’, 12.3% of Australia's population is experiencing food insecurity compared to a 7.6% average amongst other high income countries.^18^ When comparing Australia's food system scorecard to those in the global arena, it is clear that urgent action is required to contribute to global transformative efforts.

While the challenges to achieve such bold action are significant, efforts to improve our food system can have far‐reaching benefits such as improving food security and nutrition, social and gender equity, and community resilience, amongst others.^19^ In 2019, the EAT‐Lancet Commission advised that ‘nothing less than a Great Food Transformation’ is required, including a global shift towards healthy and sustainable diets.^2^ This transformation requires cross‐sectoral, global collaboration. Global targets exist to support Australia's effort to achieve this population‐wide dietary shift, including Agenda 2030 and the Paris Agreement, which are proving to be effective mechanisms for attracting political will and driving change.^20^, ^21^, ^22^ The United Nations' Decade of Action on Nutrition commits United Nations' Member States, including Australia, to implement public health policy to create sustainable, resilient food systems for healthy diets for all.^23^ It is agreed that a whole‐of‐system approach is required, as evidenced by Indigenous food systems which sustained biodiversity, natural resources and an abundance of food sources in both land and water‐based ecosystems.^24^ Broadly defined, the food system ‘encapsulates the activities, outcomes and actors involved in agriculture, storage, processing and manufacture, distribution, retail and consumption’.^25^ ^,p.1097^ The points of intersection between diet and this broader food system present opportunities to achieve systemic transformation, whereby efforts to promote the consumption of healthy and sustainable diets can trigger transformative action across the entire food system.^19^, ^26^, ^27^, ^28^

Dietitians utilise scientific principles and methods in the study of nutrition, to influence the wider food environment and ultimately affect food intake and eating behaviour.^29^ Dietitians therefore have a key role to play in contributing to food system transformation, in particular by facilitating a population‐wide shift to healthy and sustainable diets. Role statements developed in Australia and overseas describe opportunities for dietetic practice to achieve this within various settings and levels of public policy.^30^, ^31^ For example in food‐based dietary guidelines at the population‐level, food procurement and menu planning policies at an institutional‐level, product reformulation within the private food industry, nutrition education to client groups, community groups and other health professionals, and medical nutrition therapy at the group and individual level.^32^ This paper presents Dietitians Australia's position on healthy and sustainable diets based on a review of existing literature. Using this evidence, key recommendations are provided to guide future research, practice and advocacy efforts, including an investment in capacity building activities and tertiary education to further equip Australia's current and future dietetic profession to contribute to food system transformation.

## 2 METHODS

Exploration of existing literature was guided by a scoping review methodology. This approach was deemed suitable given the diverse and largely heterogenous literature available to answer the broad and complex research questions.^33^ While guidance was taken from the Preferred Reporting Items for Systematic reviews and Meta‐Analyses extension for Scoping Reviews (PRISMA‐ScR), not all checklist items were feasible for this current study.^34^ The following five‐stage approach for scoping reviews was undertaken.^35^, ^36^, ^37^

### 2.1 Stage 1: Identifying the research questions

To present an overview of existing evidence on healthy and sustainable diets, with the purpose of informing Dietitians Australia's policy recommendations, three research questions were developed by the expert working group: (i) What are the characteristics of healthy and sustainable diets? (ii) What approaches are being taken by researchers to measure health and environmental sustainability outcomes of population diets? (iii) What evidence‐based policy options exist to facilitate the uptake of healthy and sustainable diets in Australia?

### 2.2 Stage 2: Identifying relevant studies for inclusion

Searches were designed and conducted for each of these research questions respectively, drawing from both peer‐reviewed and grey literature (Data S1: Search Strategies). It was deemed essential to include grey literature, particularly for the third research question, to ensure policy documentation was considered. Incognito mode was utilised for all Google Scholar searches to ensure replicability. To restrict the size of this study, literature published in English during a defined time period as outlined in the search strategies (Data S1) was retrieved and screened against the below inclusion criteria (Tables 1, 2, 3).

**TABLE 1:** Inclusion criteria for Research Question 1

Criterion	Definition
Subject	The study must have been considered relevant to both healthy and sustainable diets. Studies were excluded if they only considered one of nutrition/health or ecology/environment.
Outcome	The study must have included a description of at least one newly defined characteristic of a healthy and sustainable diet, or a *statement*/*concept* relevant to the definition of healthy and sustainable diets.
Study	The publication must have been available in English, published on or after 2012, included adequate detail to discern relevance. Study type – any peer‐reviewed publication (Google Scholar) and systematic literature reviews (PubMed) were considered.

**TABLE 2:** Inclusion criteria for Research Question 2

Criterion	Definition
Subject	The study must have been considered relevant to both healthy and sustainable diets. Studies were excluded if they only considered one of nutrition/health or ecology/environment.
Outcome	The study must have reported upon the impact of healthy and sustainable diets, and described the approaches or metrics used to measure this impact. Hypothetical scenarios such as simulation or modelling were included.
Study	The publication must have been available in English, published on or after 2019, included adequate detail to discern relevance. Study type – any peer‐reviewed publication (Google Scholar) and systematic literature reviews (PubMed) were considered.

**TABLE 3:** Inclusion criteria for Research Question 3

Criterion	Definition
Subject	The study must have been considered relevant to both healthy and sustainable diets. Studies were excluded if they only considered one of nutrition/health or ecology/environment.
Intervention	The study must have described a policy (plan, action, intervention, initiative, activity or strategy), ideally with pre‐determined intentions (goals, objectives, targets) accompanied by a planned approach or work plan to achieve or measure the desired outcome. Ad hoc activities were included, provided they were part of a broader policy.
Setting	Any policy/intervention setting was considered – all levels of government, all institutional settings, etc.
Population	Consumers or nutrition and dietetics professionals
Study	The publication must have been available in English, published on or after 2015, included adequate detail to discern relevance. Study type – any peer‐reviewed publication (Google Scholar) and systematic literature reviews (PubMed) were considered. Hypothetical scenarios such as simulation or modelling were not included.

### 2.3 Stage 3: Selection of included studies

Titles and abstracts, or in the case of Google Scholar the titles and introductory text of the first 100 results,^38^ were screened by two researchers independently. Discrepancies were discussed and resolved by these two researchers before the identified full text papers were retrieved and assessed against the inclusion criteria (Data S1: PRISMA Flow Charts).

The final included studies were complemented by literature identified through both (i) reference lists of included studies, and (ii) by members of the expert working group, ‘Dietitians Australia's Healthy and Sustainable Diets Position Statement Working Group’. These complementary sources of grey and peer‐reviewed literature were identified by expert working group members from organisational newsletters, resource repositories (e.g. Food and Agriculture Organization's Global‐Hub on Indigenous Peoples' Food Systems: www.fao.org/indigenous‐peoples/global‐hub/en, Table: www.tabledebates.org, International Confederation of Dietetic Associations' Food Sustainability Toolkit: www.icdasustainability.org), public seminars and forums on the topic of healthy and sustainable diets and other dietetic associations' public positions on the topic such as Dietitians of Canada^30^, ^39^ and the Academy of Nutrition and Dietetics (formerly American Dietetic Association).^40^ Records identified by working group members were categorised as either grey literature or those published in a peer‐reviewed journal.

### 2.4 Stages 4 and 5: Charting the data, summarising and reporting the results

Three approaches were adopted to present the results of each research question:

1. Research Question 1: To present the characteristics of healthy and sustainable diets, a summary of the published ideas, definitions and concepts from included studies was charted as a timeline in sequential order.
2. Research Question 2: To explore the approaches taken to measure the outcomes of healthy and sustainable diets, results from included studies were synthesised and described according to the four elements of a healthy and sustainable diet as proposed by the Food and Agriculture Organization.^41^ As some included studies used approaches such as modelling to measure more than one of these four elements simultaneously, an additional category was created.
3. Research Question 3: To explore policy options available to promote the uptake of healthy and sustainable diets in Australia, three reporting methods were adopted. Firstly, a brief summary was written, using examples drawn from retrieved studies. Secondly a table of specific policy examples derived from the included studies was presented, categorised according to the facilitating setting; federal government, local government, food industry and institutional. Thirdly, an overview of policy options was organised according to the NOURISHING Framework.^42^ This framework was chosen as it is intended to organise comprehensive policy options across three domains – food environment, food system and behaviour change – to promote healthier eating.^42^

A key objective of this study was to present Dietitians Australia with key recommendations to facilitate the population‐wide uptake of healthy and sustainable diets. The recommendations were first drafted by the expert working group (authors of this paper), based on the evidence presented to answer each of this study's research questions, and Dietitians Australia's capacity to influence policy across the diverse settings. The four recommendations were reviewed by Dietitians Australia's Food and Environment interest group leadership committee, Dietitians Australia's Advocacy and Policy Advisory Committee and finally Dietitians Australia's Board of Directors to produce the position statement outlined in this manuscript.

## 3 RESULTS

### 3.1 What are the characteristics of healthy and sustainable diets?

To achieve population‐level shifts towards healthy and sustainable diets, the characteristics of the desired dietary behaviours must be clearly defined. The concept of healthy and sustainable diets is not new. At the time that the view of food and health had become medicalised, Gussow and Clancy proposed that nutrition education must be extended beyond a medical view to incorporate the impact that people's food choices were having on the environment, and therefore on the nutrition of future generations.^43^ In recent years, as the threat of climate change on food production and planetary health has gained attention, the topic of healthy and sustainable diets has created momentum amongst researchers and agencies of the United Nations. There has also been a shift in focus away from working with individuals on their dietary decision‐making processes, towards efforts which create a food system that enables healthier food options and eating patterns as the default.^44^ There is an increased appreciation of Indigenous peoples' food systems and knowledge required to maintain a balance between human–ecological interactions.^24^ It is also understood that not all healthy diets have low environmental impacts, and not all environmentally beneficial diets maximise human health.^19^, ^45^, ^46^, ^47^ Therefore, to understand the way in which a more comprehensive understanding of healthy and sustainable diets has been developed, Table 4 presents a timeline of key ideas and concepts which have been published and with direct relevance to an Australian context.

**TABLE 4:** Key published ideas and concepts to define a healthy and sustainable diet

Author	Publication title	Definition
Burlingame et al.^41^	Sustainable diets and biodiversity	This landmark definition was published in the Proceedings of the International Scientific Symposium on Biodiversity and Sustainable Diets United Against Hunger, held in Rome at FAO's Headquarters in November 2010. This symposium provided experts with a platform to reach the following consensus definition: ‘Sustainable Diets are those diets with low environmental impacts which contribute to food and nutrition security and to healthy life for present and future generations. Sustainable diets are protective and respectful of biodiversity and ecosystems, culturally acceptable, accessible, economically fair and affordable; nutritionally adequate, safe and healthy; while optimising natural and human resources.’^41^ ^,p.7^ This definition continues to be referred to, for example in FAO's (2021) report and white paper on Indigenous peoples' food systems.^1^, ^24^
Friel et al.^48^	Towards healthy and sustainable food consumption: an Australian case study	Friel et al. synthesised publicly available evidence on the environmental impact of diets and defined ‘three over‐arching principles: (i) any food that is consumed above a person's energy requirement represents an avoidable environmental burden in the form of greenhouse gas emissions, use of natural resources and pressure on biodiversity; (ii) reducing the consumption of discretionary food choices, which are energy‐dense and highly processed and packaged, reduces both the risk of dietary imbalances and the use of environmental resources; and (iii) a diet comprising less animal‐ and more plant‐derived foods delivers both health and ecological benefits.’^1^, ^24^, ^48^ ^,p.1159^ Based on these, they constructed a weekly healthy and sustainable food basket, as a method with which to assess the availability and affordability of a healthy and sustainable diet.
Johnston et al.^49^	Understanding sustainable diets	Johnston et al. contribute a descriptive analysis of the determinants and processes that influence diets and their impact on health, food security and environmental sustainability. They identify five categories which determine the *sustainability* of a diet: (i) agriculture, (ii) health, (iii) sociocultural, (iv) environmental, and (v) socioeconomic.
Nelson et al.^50^	Alignment of healthy dietary patterns and environmental sustainability: a systematic review	Nelson et al. updated the systematic review conducted by the 2015 Dietary Guidelines Advisory Committee. Adding to the original 15 studies, an additional eight studies were analysed to conclude that ‘a dietary pattern higher in plant‐based foods (e.g., vegetables, fruits, legumes, seeds, nuts, whole grains) and lower in animal‐based foods (especially red meat), as well as lower in total energy, is both healthier and associated with a lesser impact on the environment’.^50^ ^,p.1005^ This was consistent with several well‐categorised dietary patterns, including vegetarian diets, US dietary guidelines‐related diets, Mediterranean‐style diets and the Dietary Approaches to Stop Hypertension (DASH) diet.
Von Koerber et al.^51^	Wholesome nutrition: an example for a sustainable diet	Von Koerber et al. described the concept of *Wholesome Nutrition* as it was developed in the 1980s in alignment with health, ecological, economic, social and cultural dimensions of nutrition. They analysed the food supply chain at all stages from production to waste disposal and identified seven principles of sustainable nutrition: (i) preference of plant‐based foods, (ii) organic foods, (iii) regional and seasonal products, (iv) preference of minimally processed foods, (v) fair trade products, (vi) resource‐saving housekeeping, and (vii) enjoyable eating culture.
Monteiro et al.^52^	The United Nations Decade of Nutrition, the NOVA food classification and the trouble with ultra‐processing	This commentary piece introduced the NOVA system of food classification, which categorises all food into four groups, based on the nature, extent and purpose of food processing. Group 1: unprocessed or minimally processed foods (e.g. seeds, fruits, leaves, stems, eggs, milk). Group 2: processed culinary ingredients (e.g. oils, butter, sugar, salt). Group 3: processed foods (e.g. bottled vegetables, canned fish, fruits in syrup, cheese, bread). Group 4: ultra‐processed foods (e.g. soft drinks, sweet or savoury packaged snacks, reconstituted meat products, pre‐prepared frozen dishes). Monteiro et al. (2018) described the rapidly increasing production of ultra‐processed products, which contribute to climate disruption, pollution, degradation and depletion of air, land, water and sources of energy, as a world crisis to be addressed as part of the United Nation's Sustainable Development Goals.
World Health Organization (WHO)^53^	A healthy diet sustainably produced (information sheet)	WHO identified that to ensure a healthy diet for current and future populations, we must 'focus on the most vulnerable populations, on promoting a healthy and diverse diet and on changing to sustainable food production systems’.^53^ ^,p.3^ WHO presented 14 recommendations for a healthy diet that is sustainably produced, which are applicable to low‐, middle‐ and high‐income country settings with consideration of health, environmental, gender, and equity outcomes.
EAT Lancet Commission^2^	Food planet health—summary report	The EAT Lancet Commission defined the planetary health diet as a means to nourish a population of 10 billion people by 2050 while respecting planetary boundaries. This *flexitarian diet* is ‘largely plant‐based but can optionally include modest amounts of fish, meat and dairy foods.’^53^ ^,p.32,p.11^ The EAT‐Lancet Commission defined scientific targets for a planetary health diet for an intake of 1500 kcal/day however encourage local interpretation to reflect the culture, geography and demography of the population and individuals.
Smetana et al.^54^	A path from sustainable nutrition to nutritional sustainability of complex food systems	Smetana et al. describe *Nutritional Sustainability* as a concept which ‘sets environmental sustaining capacity as a baseline level for balanced nutrition’ while also aiming for the ‘search of food system driving nodes’.^54^ ^,p.39^ *Nutrition Sustainability* does not aim for the support of solutions of producing enough or more food for increasing population (*sustainable nutrition*), neither does it contradict other similar concepts [*sustainable nutrition security*, *nutritional life cycle assessment (LCA)*].^54^
Lawrence et al.^55^	Sustainable, resilient food systems for healthy diets: the transformation agenda	Lawrence et al. commented on the EAT Lancet Commission's planetary health diet by summarising the recommendations, commending the comprehensive approach taken and outlining the response amongst key stakeholders. Some criticisms presented which are of relevance to defining healthy and sustainable diets are those concerning nutritional adequacy for population subgroups such as pregnant women, the feasibility of uptake given the flexitarian diet is largely prescriptive and the omission of ultra‐processed foods in their analysis.
FAO and WHO^56^	Sustainable healthy diets – guiding principles	This document resulted from an international expert consultation to develop guiding principles for sustainable healthy dietary patterns to be translated into policy action. Five background papers were prepared by global experts in advance of a three‐day consultation in Rome. They defined sustainable healthy diets as those which ‘achieve optimal growth and development of all individuals and support functioning and physical, mental, and social wellbeing at all life stages for present and future generations: contribute to preventing all forms of malnutrition (i.e. undernutrition, micronutrient deficiency, overweight and obesity); reduce the risk of diet‐related non‐communicable diseases; and support the preservation of biodiversity and planetary health.’^56^ ^,p.11^ There are 16 guiding principles which relate to the health, environment and sociocultural aspect of sustainable healthy diets.
Kuhnlein et al.^57^	Indigenous food systems: contributions to sustainable food systems and sustainable diets. In Burlingame B. and Dernini S., Sustainable Diets. Linking Nutrition and Food Systems.	Kuhnlein et al. describe that Indigenous peoples' have ‘a collective experience in managing 22% of the world's ecosystem and land mass and preserving the majority of the planet's biodiversity. Indigenous peoples understand how their local foods are resilient and adapted to their local environments, even when climate challenged. They know the animals and plants that are natural resources in the world's forests, pastures, riverine lands and waters, lakes, and seas, which contain the genetic material of the world's biodiversity. The knowledge of these resources is grounded in their culture, spirituality and historical legacy. Those who can relate and express such knowledge can help the world to develop, realise and enjoy the benefits of Indigenous food systems, which are essential for sustainable diets’, whilst safe‐guarding the rights of Indigenous peoples.^57^ ^,p.67^
Barbour et al.^58^	Translating evidence into policy action: which diet‐related practices are essential to achieve healthy and sustainable food system transformation?	Barbour et al. sought to define healthy and sustainable diet‐related practices that could be targeted by policy‐makers. These diet‐related practices were defined as the activities that an individual engages in to source, store, prepare, consume and dispose of food. A review of relevant United Nations' publications dated after FAO's (2012) landmark definition, distilled 13 commonly recommended healthy and sustainable diet‐related practices: (i) select food grown using sustainable food production practices, valuing and respecting Indigenous knowledges, (ii) strengthen local food systems by connecting with primary producers, (iii) eat seasonally, incorporating native and wild‐harvested foods, (iv) eat locally available foods, (v) avoid over‐consumption beyond energy requirement, (vi) consume no more than recommended animal‐derived foods, (vii) limit intake of ultra‐processed, nutrient‐poor and over‐packaged food, (viii) increase intake of plant‐based foods, (ix) eat a wide variety of foods to promote biodiversity, (x) adopt food waste‐minimisation strategies, (xi) preference home‐made meals and share with others, (xii) consume safe tap water as preferred drink, and (xiii) breastfeed infants where possible.

Scholars with expertise in healthy and sustainable food systems agree that 'no single exemplar diet exists’.^45^ ^,p.132^ However, as outlined in Table 4, contributions to defining a healthy and sustainable diet have remained consistent with the FAO's (2012) original definition in that four elements must exist; a healthy and sustainable diet must (i) be nutritionally adequate, healthy and safe, (ii) have low environmental impact and be protective of natural resources and biodiversity, (iii) be culturally acceptable and (iv) be accessible, economically fair and affordable.^45^

### 3.2 What approaches are being taken by researchers to measure health and environmental sustainability outcomes of population diets?

To assess the quality of population diets against these four elements of a healthy and sustainable diet, scholars have adopted a range of methods, such as modelling, life cycle assessment, and land use analysis and applied multiple disciplinary lenses.^50^ Before presenting the approaches taken by scholars within the peer‐reviewed literature, it must be acknowledged that Indigenous peoples' food systems have sustained life since millennia based on ‘keen [detailed, complex and sustained] observations of the processes and effects of nature’,^24^ ^,p.6^ that may not yet be evident in the conventional hierarchies of scientific evidence.^59^

In looking at the first element, *the nutritional adequacy*, *health and safety of a diet*, Kumanyika et al. compared three approaches to measuring the healthiness of diets to inform the expert consultation on Sustainable Healthy Diets; WHO dietary guidelines, Global Burden of Disease risk factors and analysis of whole dietary patterns with health outcomes in population studies and clinical trials.^60^ Although interested solely in health outcomes, they identified crucial win–win opportunities to promote healthy and sustainable diets by increasing plant‐based foods, reducing processed meats, limiting the intake of salt, free sugars, saturated fats, trans fats and industrially processed foods.^60^ Another approach to assess the nutritional quality of population‐level diets is the NOVA classification system referred to in Table 4.^61^ Nine systematic reviews have synthesised a substantial body of evidence reporting associations between ultra‐processed foods and a range of adverse health outcomes.^62^, ^63^, ^64^, ^65^, ^66^, ^67^, ^68^, ^69^, ^70^ Machado et al. found a positive linear trend between high intake of ultra‐processed foods and high intake of nutrients linked to non‐communicable diseases amongst Australian adults.^71^ Ultra‐processed foods have also been shown to have an association with adverse sustainability outcomes.^72^

In considering the second element, *environmental impact of a diet*, an integrative review of the metrics in use identified that journal literature mostly addressed greenhouse gas emissions and, to a lesser extent, land and water use.^73^ This review identified that soil carbon stocks were overlooked as an important indicator of environmental impact.^73^ This represents a misalignment in the scientific methodologies between publications aiming to inform climate action through agricultural production and those assessing the environmental impacts of foods to define a healthy and sustainable diet.^73^ Chai et al. conducted a systematic review to compare vegan, vegetarian and omnivorous diets for their environmental impact.^74^ They measured the environmental impact based on greenhouse gas emissions, land use and water footprint.^74^ Focusing on the Swedish population diet, Mehlig et al. examined the changes in diet‐related greenhouse gas emissions since the turn of the century, demonstrating the use of this single measure of sustainability to provide insights to a trend over time.^75^

Moving to Australian research on this environmental impact element, Ridoutt et al. quantified the water scarcity footprint of 9341 Australian adults using dietary intake data and compared this to the planetary boundary for freshwater use.^16^ They concluded that diets based on Australia's dietary guidelines are within the planetary boundary for freshwater.^16^ Using the same dietary intake data, Ridoutt et al. also investigated use of a weighted environmental impact score, factoring in climate footprint, water‐scarcity footprint and cropland‐scarcity footprint. Upon considering wider impacts, they concluded that a diet based on Australia's dietary guidelines could achieve a lower environmental impact score, but not low enough to achieve planetary boundary targets due to the impacts of the food production system.^76^ Forbes et al. conducted a rapid review to determine the environmental impacts associated with food consumption in Australia and New Zealand and of the 20 studies included, greenhouse gas emissions (*n 12*) were the most commonly used environmental indicator followed by water use and environmental footprint (*n 7*) and carbon footprint (*n 3*).^77^ Shamsi et al. explore the rich legacy of Aboriginal fishing cultures in Australia, offering valuable lessons to conserve aquatic resources and understand human–ecology interconnectedness.^78^ Their literature review describes examples of fishing practices, ideology and sustainable philosophy, such as only taking enough fish to nourish individuals and communities, and restricting fishing based on seasons and stock abundance, explaining how these are measured.^78^

To assess the *cultural acceptability of a diet*, Hachem et al. reviewed the contribution of two territorial diets, the Mediterranean and New Nordic diets, towards environmental, sociocultural and economic sustainability outcomes, arguing that some territorial or ‘regional’ diets can be a catalyst for food systems transformation.^79^ Although not technically assessing the cultural acceptability, various socio‐cultural drivers have likely contributed to the increased intake of ultra‐processed foods.^80^ For example, Baker et al. suggest that inequitable gender distribution of household work including shopping for and preparing family meals, may explain the higher proportions of semi‐prepared, ready to eat ultra‐processed meals and snacks consumed in Australia and other high‐income countries.^80^

And finally, in assessing the *economic accessibility and affordability of a diet*, the use of a hypothetical food basket is a commonly used method to assess and monitor food availability and cost internationally and within Australia.^81^, ^82^, ^83^ In measuring the affordability of a healthy and sustainable diet in Australia, Barosh et al. compared the price of a typical food basket with a healthy and sustainable food basket within five neighbourhoods of Greater Western Sydney.^48^, ^84^ They concluded that those most economically disadvantaged, both at a neighbourhood and household level, experience the greatest inequality in affordability of the healthy and sustainable diet. More recently, Goulding et al. compared an Australian‐specific planetary health diet basket modelled on the EAT‐Lancet Commission's recommended diet, to the typical Australian diet basket for two adults and two children.^85^ Analysing the cost of each basket in low, medium and high socio‐economic areas in each Australian state and territory identified that the Planetary Health Diet was more affordable for Australians living in Metropolitan areas than the typical Australian diet.^85^ In another approach to measure the affordability of a healthy and sustainable diet, Donati et al. assessed dietary intake data from 104 young adults in Italy to identify two diets; the minimum cost healthy diet and the most environmentally sustainable diet (based on carbon emissions, water consumption and land use).^86^ They then integrated both economic and environmental objectives to define a healthy, sustainable and affordable diet for this population group.^86^

To assess multiple outcomes of a population diet simultaneously, benchmark modelling has been shown to be useful, whereby pre‐determined diets are evaluated against indices to quantify health, environment and affordability outcomes. For example, Mertens et al., compared a diet which adhered to food‐based dietary guidelines in Denmark, Czech Republic, Italy and France and evaluated these according to their nutritional adequacy using the Nutrient Rich Diet score and their greenhouse gas emissions.^87^ Their modelling measured three preferential diet scenarios; dietary preferences, nutrient quality and environmental impact and they concluded that fully maximising health and minimising greenhouse gas emissions cannot be achieved simultaneously.^87^ Chen et al. conducted a multi‐dimension, multi‐indicator analysis involving nine alternative dietary scenarios, three nutritional quality scores, five environmental indicators, one economic measure and one human health indicator.^88^ They concluded that transition towards a healthy diet (dietary guidelines of Swiss Society for Nutrition) was the most sustainable option and would result in 36% lesser environmental footprint, 33% lesser expenditure and 2.67% lower adverse health outcome (DALYs) compared with the current diet.^88^

Wrieden et al. developed a framework to quantify health, affordability and environmental sustainability measures for food purchase survey data in the United Kingdom.^89^ They applied a life cycle assessment approach to detailed food composition data, measuring greenhouse gas emission, land use, diet quality (based on dietary guidelines and food cost), all standardised according to household income.^89^ Allen et al. adopted a Delphi survey to propose a new metric system (with 18 indicators) to assess the sustainability of food systems and diets, specific to the Mediterranean area.^90^ They also considered affordability and health in their sustainability assessment.^90^ Clark et al. measured five health outcomes (type 2 diabetes, stroke, coronary heart disease, colorectal cancer and mortality, and five environmental outcomes: greenhouse gas emissions, land use, scarcity‐weighted water use, acidification and eutrophication) to conclude that dietary transitions towards healthy food consumption will generally also improve sustainability.^91^

Springmann et al. conducted a modelling study to assess the healthiness and sustainability of national and global food based dietary guidelines of 85 countries.^92^ They concluded that adoption of the EAT‐Lancet's planetary health diet as compared to the WHO's dietary guidelines would achieve a 34% reduction in premature mortality and reduce greenhouse gas emissions by more than three times.^92^ Of the 85 countries' dietary guidelines assessed, most (67%–87%) were incompatible with meeting targets set within the Paris Climate Agreement.^92^ Hence, to achieve ambitious targets set to nourish a growing global population within planetary boundaries, national dietary guidelines must be both healthier and more sustainable. Springmann's earlier work was analysed as part of a systematic review by Jarmul et al. which assessed the available published evidence on the effect of ‘sustainable diets’ (typically high in plant‐sourced and low in animal‐sourced and processed foods) on environmental footprints and human health.^93^ Analysis of 18 studies was conducted using six environmental outcomes and seven health outcomes, which consistently placed a ‘sustainable diet’ as having both positive health effects and reduced environmental footprints with the exception of increased water use.^93^ They discuss the consideration that co‐benefits are not universal, with trade‐offs required across the health and environmental measures even when population diets are carefully designed, evidence based and adapted to contextual factors.^93^ Bunge et al. conducted a systematic review to explore the existing food profiling models as a tool to inform the development of food labels that account for nutrition and environmental sustainability.^94^ Published in the Lancet Planetary Health, their review identified 16 sustainable food profiling models from which they describe a number of advantages and disadvantages.^75^ Amongst the disadvantages, few profiling models to date account for at least two environmental impact factors and even less included other dimensions of sustainability or nutrition measures.^75^

Within an Australian context, Hendrie et al. modelled the average adult diet against dietary patterns consistent with Australia's dietary guidelines and identified that a 25% reduction in greenhouse gas emissions would result if our population were to follow existing guidelines.^15^, ^46^ Also modelling Australian dietary patterns, Candy et al. compared a *healthy mixed diet*, with both animal and plant foods, to a *healthy plant‐based diet, with only p*lant foods.^95^ Both diets met Australian dietary guidelines and four sustainability principles; avoiding over‐consumption, reducing intake of discretionary foods, reducing animal products, and reducing food waste.^95^ Modelled outcomes included food availability, water use, land use, greenhouse gas emissions, fuel and energy use and fertiliser use, and identified that a population‐wide shift towards the plant‐based diet would be associated with less environmental impact, however fertiliser use and land availability concerns would need to be addressed.^95^

As described, various approaches exist to measure the health and environmental outcomes of healthy and sustainable population diets. Madzorera et al. highlighted a number of challenges for researchers and practitioners working to measure healthy and sustainable diets, including the lack of standardisation and validation of diet quality (health and sustainability metrics) for countries globally.^96^ They acknowledged that metrics must also consider convenience, preference and desirability which influence food choices.^96^ In summary, further research and development of diet‐related heath and sustainability metrics is required. These metrics must consider all four elements of FAO's (2012) definition of a healthy and sustainable diet to support effective measurement and population‐wide uptake of this diet.

### 3.3 What evidence‐based policy options exist to facilitate the uptake of healthy and sustainable diets in Australia?

Having described progress to define what a healthy and sustainable diet is and how it is measured, this section explores approaches to facilitate the uptake of these diets in Australia. Peer‐reviewed literature continues to emerge, demonstrating both the effectiveness and feasibility of various approaches. Bene et al.^97^ defined five areas of research and action required to operationalise the EAT‐Lancet Commission's recommendations; economic viability (e.g. discounts for low‐income households to purchase fruits and vegetables), political economy (e.g. accountability mechanisms to ‘fame and shame’ actions taken by powerful food actors), cultural norms (e.g. guiding consumer behaviour by altering the choice architecture of the food environment), equity (e.g. full supply chain traceability to discourage child labour), governance and tools (e.g. use of foresight techniques to inform policy and decision‐making).^97^ Parsons and Hawkes sought to identify food systems policy with the co‐benefits of healthy, environmental and economic policy goals in the United Kingdom.^98^ They identified six aspects of food systems that show potential for all three outcomes to come together; public procurement, a Common Agricultural Policy, school fruit and vegetable schemes, investing in small and medium‐sized enterprises and entrepreneurship, short supply chains and building skills.^27^ Also from the United Kingdom, the Behaviour Insights team presented a range of strategies which target four key stakeholder groups (sustainability leaders, consumers/citizens, the food industry and governments), and reflect three critical pillars of dietary change; making sustainable food more appealing, normal and easy.^99^

In considering which strategies are most feasible to achieve a widespread shift to sustainable diets, we must first understand the drivers for change. One such driver is the habitual behaviour embedded in individuals over time and the cultural and social norms that support these habits.^97^ Eker et al. used an integrated assessment model to demonstrate this social norm effect, whereby, for example, high vegetarianism amongst a population will accelerate a further shift to a vegetarian diet amongst others in this same population group.^100^ This example supports the need for investment in strategies that are motivated by either intrinsic identity or by group dynamics, to effectively achieve this population‐level shift towards healthy and sustainable diets.^100^ Importantly, the food system must support these social changes throughout the food supply chain, beyond just the consumption phase. For example, if strategies to trigger this social norm effect are successful in promoting a reduced intake of meat, the system must offer adequate sustainably grown and processed plant‐based protein options. With many strategy options being discussed, it is vital to also consider available mechanisms to ensure best‐practice evidence is translated into policy decision‐making. An example of such a mechanism, the Food Systems Dashboard, was created in 2020 to visually present curated data from public and private sources to identify context‐specific levers for change to inform policy decision‐making at a country level.^101^ Lawrence et al. developed a policy formulation tool to strategically inform food and nutrition policy that aims to promote healthy and sustainable diets.^102^ They present an ‘Orders of Food Systems Change’ schema, intended to guide policymaking through (i) first order change related to *adjusting* individual components of the food system to improve their performance efficiency, (ii) second order change related to *reforming* interaction between and within the inter‐related components of the food system and (iii) third order change which focuses on *transforming* the orientation of the food system as a whole, so that it becomes a tool to facilitate healthy and sustainable diets.^102^

In reviewing current evidence about the efficacy of various policy options to facilitate the uptake of healthy and sustainable diets, the policies presented in Table 5 were identified. In addition to these policy options, dietary guidelines are an effective mechanism to promote healthy and sustainable diets.^92^, ^146^ Dietary guidelines are implemented at a Federal Government level and serve as an evidence‐informed reference standard to guide policy activities and inform research into population diets.^146^

**TABLE 5:** Settings and policy options which facilitate population‐wide uptake of healthy and sustainable diets

Setting	Policy	Examples in the literature (countries where the example is drawn from)
Federal government	National food and nutrition strategy	National Food Strategy (United Kingdom)^103^, ^104^ Australia's attempt to create a National Food Plan (Australia)^105^
	Taxation	Tax on sugar sweetened beverages and ultra‐processed foods (Australia)^106^
Local government	Governance	Engagement from Indigenous leaders in policy development (International)^24^ Policy and planning to influence local food environments (Australia)^107^ Food policy coalitions as a means to influence local food environments (Australia)^108^ Case study of Europe's ‘Common Food Policy’ to demonstrate how governance reforms can trigger a shift to healthy diets and sustainable food systems (Europe)^109^ City‐region food system framework (International)^110^ Madrid – Role of cities in food governance (Spain)^111^ Co‐developing (local government and key stakeholders) an indicators toolbox for action to support urban cities to evaluate performance according to food system sustainability (United Kingdom)^112^
	Modifying the local food environment	Local government‐led strategies as part of the Milan Urban Food Policy Pact (International)^113^, ^114^ Food system sustainability from local to global approach (International)^115^ Participatory food policy‐making process – Australian case study (Australia)^116^ Shaping physical, economic and policy components of food environments for healthy and sustainable diets (International)^44^ Benchmarking as a public health strategy to create healthy food environments – evaluation of INFORMAS (International)^117^ Improving food environments using INFORMAS (New Zealand)^118^ Multi‐sector participatory approach working with community stores to enhance food security in remote Indigenous communities (Australia)^119^ Including alternative food networks (e.g. non‐supermarket retail options and civil society groups) in urban policy (Italy)^120^
Food industry	Re‐orientation of the retail food environment	Restricting the merchandising of discretionary food and beverages in retail settings (Australia)^121^ Barriers and facilitators for creating healthy food retail outlets – perspectives from Australian local government authorities (Australia)^122^ Applying the ecological determinants of health perspective to reconsider the current retail foodscape (United States of America and Canada)^123^ A systematic review of factors influencing sustainable food consumption behaviours amongst university students, examining the effects of choice architecture interventions (International)^124^
	Food labelling	A systematic review comparing consumer preference for nutrition, environmental and social responsibility food labelling (International)^125^ A systematic review of sustainable food profiling models used to inform the development of food labels accounting for both nutrition and the environment (International)^94^ A systematic review and meta‐analysis exploring the willingness to pay more for foods with environmental sustainability labels (International)^126^ Consideration of the practicalities of labelling to encourage sustainable food choices by consumers and trigger systemic changes (International)^127^, ^128^, ^129^, ^130^ Traffic light labelling of meal choices as a method of persuasion (United Kingdom)^131^
Institutional	Institutional food service guidelines and auditing	A systematic review of hospital food service; environmental and associated economic impacts, outcomes of strategies aiming to improve sustainability and perspectives of patients, staff and stakeholders about these strategies (International)^132^ A systematic review exploring consumer expectations and responses towards environmentally sustainable initiatives of foodservice operations (International)^133^ A systematic review of food waste audit methods in hospital food services – development of a consensus audit tool (International)^134^ Developing and implementing national‐level healthy and sustainable guidelines in America for institutional food service settings (United States of America)^135^, ^136^ Victoria's guidelines for healthy and high‐quality food in public hospitals and aged care facilities (Australia)^137^
	Food procurement	New York's public food procurement policies (United States of America)^138^ Copenhagen organic conversion in public kitchens (Denmark)^139^ Public food procurement as a policy instrument to address social, economic, environmental, health and nutrition outcomes by promoting sustainable food systems and diets (Brazil, Paraguay and United States of America)^140^
	Menu adaptation	Meatless Monday in Armed Forces (Norway)^141^ Evaluation of meatless Monday in a National school meal program (United States of America)^142^ Impact of offering more vegetarian cafeteria meal options (United Kingdom)^143^ Improved school meals (health and environmental sustainability) using linear optimization, without negative effects on food waste, consumption or cost (Sweden)^144^ Randomised controlled field experiments nudging conference participants to select a vegetarian default lunch option (Denmark)^145^

Springmann et al. identified from their modelling of 85 countries that the adoption of food‐based dietary guidelines improved health outcomes and reduced greenhouse gas emissions.^92^ Their analysis however suggested that national guidelines could be healthier and more sustainable, in particular limiting the consumption of animal‐derived foods, increasing plant‐based foods and wholegrains and attaining a balanced energy intake.^92^ Reinhardt et al. conducted a systematic review of the evidence on dietary patterns and sustainability in the United States of America (USA), by comparing the sustainability of diets adhering to the dietary guidelines with current diets.^147^ Their results challenged prior findings that diets adhering to the dietary guidelines were more sustainable than average diets, indicating that the USA dietary guidelines may lead to similar or increased greenhouse gas emissions, energy use, and water use compared to the current diet in the USA.^147^ Ritchie et al. assessed the implications of a number of dietary guidelines including Australia's on greenhouse gas emissions and identified them to be highly inconsistent with the Paris Agreements' 1.5°C global warming target.^148^ In comparing the average Australian intake to Australia's dietary guidelines, Hendrie et al. concluded that to promote health and environmental sustainability, dietary guidelines must facilitate a reduction in non‐core foods and the consumption of no more than recommended serves of core food items, especially red meat.^15^ With processed and ultra‐processed food intake increasing globally, the NOVA categorisation framework is a recommended tool to inform the development of both healthier and more environmentally sustainable guidelines.^52^, ^72^, ^80^ In assessing if and how environmental sustainability messages are included in food‐based dietary guidelines, Fischer and Garnett identified firstly that only 83 of a possible 215 countries had official guidelines.^146^ And of those with guidelines, only four countries at the time of this review had included sustainability messages; Brazil, Sweden, Qatar and Germany; and two countries, United States of America and Australia, had attempted to include them however failed to achieve government endorsement.^146^

As dietary guidelines are periodically reviewed by federal governments globally, it is essential that evidence on both health and sustainability is considered, and that this evidence considers biases involved in industry‐funded studies. In looking to best‐practice examples of dietary guidelines with prominent sustainability messages, scholars have published the evidence used to support Brazil's National Dietary Guidelines^149^ and the development of Netherlands' healthy and sustainable food‐based dietary guidelines.^150^ Dietary guidelines are not only a tool to provide advice to the general public on foods, food groups and dietary patterns, they also form the basis for food policy decision‐making and are therefore fundamental to shift consumption patterns in healthier and more environmentally sustainable directions.^150^ As outlined in Table 5, there are a number of settings and policy options to facilitate the population‐wide uptake of dietary guidelines.

## 4 RECOMMENDATIONS

This position paper presents an overview of the current evidence to define and measure healthy and sustainable diets, and an overview of policy options to facilitate the uptake of healthy and sustainable diets. The NOURISHING Framework is intended to organise comprehensive policy options across three domains – food environment, food system and behaviour change – to promote healthier eating.^47^ Table 6 presents an overview of existing policy options to facilitate the uptake of healthy and sustainable diets, as presented in the current literature, organised according to the NOURISHING Framework.^47^

**TABLE 6:** Policy options to promote healthy and sustainable diets

Domain	Policy area^47^ (NOURISHING framework)	Policy recommendation	Policy leadership
Food environment	*N*utrition label standards and regulations on the use of claims and implied claims on food	Integration of ecological principles in federally implemented, mandatory interpretive front‐of‐pack food labelling schemes Warning labels on menus and displays in out of home venues (e.g. Carbon Footprint metrics, Ultra‐Processed Food Advisory Statement)	Federal government Retail/hospitality setting
	*O*ffer healthy food and set standards in public institutions and other specific settings	Procurement policies for public and private food service facilities (e.g. health services, prisons, aged‐care, childcare, supported residential services) Adequately resourced measures to reduce and reuse commercial and domestic food‐related waste in line with circular economy principles (e.g. domestic and hospitality composting options) Mandatory standards for food available in schools and other learning institutions (canteens and vending machines) and in their immediate vicinity	State government (to be implemented by local government) Department of Health Department of Education School communities
	*U*se economic tools to address food affordability and purchase incentives	Tax on sugar sweetened beverages and nutrient‐poor ultra‐processed foods Tax on foods produced using production practices which deplete natural resources, to incentivise demand for foods produced using regenerative production practices Targeted subsidies/discounts on locally produced foods	Federal government Independent retailers
	*R*estrict food advertising and other forms of commercial promotion	Restricted marketing of discretionary and ultra‐processed foods to children (e.g. television, sports club sponsorships, posters on public transport, written and online communication) Restricted marketing strategies (price, placement, product, promotion) of discretionary and ultra‐processed foods in retail settings	Federal government
	*I*mprove nutritional quality of the whole food supply	Reformulation of food products to prioritise less processed options (e.g. using the NOVA Framework to incentivise food manufacturers to focus on groups one, two and three and minimise the production of group four, the ultra‐processed foods)	Food industry
	*S*et incentives and rules to create a healthy retail and food service environment	Ensuring urban planning legislation/requirements allow for equitable access to healthy and sustainable food (e.g. zoning regulations to prioritise farmers markets, green grocers, social solidarity supermarkets, bulk food stores over retail outlets selling fast food and ultra‐processed foods) Incentivise commercial kitchens (e.g. cafes and restaurants) to offer healthy and sustainable menu options by rewarding and promoting their efforts	Local government
Food system	*H*arness supply chain and actions across sectors to ensure coherence with health	Invest in governance structures to engage multi‐sectorial stakeholders including the community from across the food supply chain in healthy public policy (e.g. regional food networks, food policy coalitions, collective impact approaches) Use public procurement policies to increase accessibility to fair trade, organic, locally produced (where planetary boundaries are respected) foods, ethically sourced Indigenous foods and food produced through regenerative agricultural practices Work across all institutional settings to change food service provision (e.g. less animal‐derived foods and more plant‐based proteins on the menu, efforts to minimise food‐related waste) to support social normative shifts Support urban agriculture in health and planning policies Implement sustainable soil management practices including measurement of carbon stocks to support growth of plant foods with optimal nutritional value reduce atmospheric carbon.	Local, state and federal governments Food industry Key stakeholders from throughout the food supply chain Federal government
Behaviour change communication	*I*nform people about food and nutrition through public awareness	Integrate sustainability principles within future iterations of Australia's National dietary guidelines Reorient the dietetics workforce to be equipped with skills and knowledge to influence action across the food system and contribute to these recommendations Regular review of role statements to inspire and support nutritionists and dietitians to contribute to food system transformation Public awareness campaigns and social marketing interventions to promote healthy and sustainable diet‐related practices	Federal government International Dietitians Australia State governments
	*N*utrition advice and counselling in healthcare settings	Training resources to enable the existing nutrition and dietetics workforce to implement healthy and sustainable food policy across various settings and areas of practice (e.g. British Dietetic Association's One Blue Dot program, training on healthy and sustainable food procurement and food service practices) Professional development opportunities to equip nutritionists and dietitians to provide sound nutrition advice and counselling which considers the ecological outcomes of dietary advice Development of education material and consumer resources for the general public on healthy and sustainable diets Mandatory tertiary education on food sustainability for the future nutrition and dietetics workforce education^32^, ^151^ (e.g. competency standards, stand‐alone units/modules relevant to the various areas of dietetic practice, integration of sustainability evidence and principles across the entire curricula)	Dietitians Australia Accreditation bodies Tertiary institutions
	*G*ive nutrition education and skills	Professionals involved in delivering nutrition education and skills must consider the environmental impact of dietary behaviours, across the diverse practice areas (e.g. hospitals, health services, aged care, food industry, primary production, schools, early learning centres, recreation and community centres) Training for stakeholders from the hospitality, food procurement and food service industries (e.g. caterers and food service providers) in incorporating sustainability principles into food procurement, food service practices and menus Dietitians and Nutritionists working within the food industry can influence practices throughout the supply chain (e.g. procurement, manufacturing, distribution, packaging to increase public accessibility to healthy and sustainable diets	Healthcare professionals Hospitality, food procurement and food service Industries Food industry Food supply stakeholders

Dietitians have an important role to play in contributing to global sustainable development targets, specific to and beyond efforts to transform our current food system.^20^, ^21^ This contribution relies on inter‐sectoral collaboration, particularly with those required to undertake the most significant changes such as our food industry and the agricultural sector. The perspectives and expertise of Indigenous peoples is critical to effective governance and policy‐making related to healthy and sustainable food systems. Based on Dietitians Australia's expertise, capacity to influence action and positioning within this policy area, the following four recommendations have been prioritised.

1. The development of a comprehensive, adequately resourced National Food and Nutrition Strategy which honours Indigenous knowledges on food systems, detailing a strategic plan to improve health, equity and sustainability outcomes of our food system (including policy options as presented in Table 6).
2. The prominent integration of ecological sustainability principles in the next iteration of Australia's dietary guidelines, to foster a population‐wide demand for healthy and sustainable food to trigger and support change across the whole food system.
3. The reorientation of our food environment to prioritise access to healthy and sustainable dietary food options, including (i) a food labelling scheme which integrates health and ecological outcomes, (ii) settings‐based approaches (e.g. food procurement policies for food services, retail and public facilities) and (iii) supportive federal and state policy to facilitate local government action.
4. Investment in capacity building activities for our current and future nutrition and dietetics workforce, including more opportunities for Aboriginal and Torres Strait Islander peoples to contribute to food system transformation through collective partnerships, effective tertiary education and continuing professional development.

It is critical that the opportunity is prioritised for Aboriginal and Torres Strait Islander People to be involved in the further development and translation of these recommendations into practice and policy.

## 5 FUTURE RESEARCH OPPORTUNITIES

This position paper presents an overview of the current literature relevant to each of the three defined research questions, to inform the development of policy recommendations for Dietitians Australia and its members. The methods used to develop this position paper were designed within the scope of this project and have identified that future iterations of this position paper should be founded on traditional systematic or scoping review methodology.

This position paper identified some specific opportunities for future research. For example, as described by Lang, current evidence on sustainable diets suggests a need to shift the focus away from simply producing more food – the productionist mid‐20th century policy vision ‐ towards changing the *what* and *how* food is produced and consumed.^152^ Ridoutt and Huang advise that research must prioritise efforts to demonstrate the environmental impact of ultra‐processed and non‐core foods.^76^, ^153^ This evidence has largely been overlooked in current reviews which have focussed on meat, dairy and agricultural practices.^153^

In terms of dietetic involvement in this research, a Delphi study identified that *food systems*, *health and nutrition promotion with the inclusion of planetary health and sustainability perspectives* should be considered a research priority for our profession over the coming decade.^154^ In developing this position paper, it is clear that dietitians can continue to contribute to research which will inform evidence‐based policy action, as recommended in this position paper. For example, dietitians can add to current research to identify opportunities and challenges to influence food environments in the settings in which they work, including reforms to food procurement, food service and food retail settings policies and practices, and influencing the food supply from within the food sector. Another example, is to consider how healthy and sustainable diets can be promoted through effective food governance and collaboration on key policy interventions such as Australia's National Preventative Health Strategy and the National Obesity Strategy.^155^

## CONFLICT OF INTEREST

Liza Barbour, Ellyn Bicknell, Stefanie Carino, Molly Fairweather, and Julie Brimblecombe are members of Dietitians Australia. Elizabeth World is a staff member of Dietitians Australia. Mark Lawrence is a representative of Dietitians Australia's Advocacy and Policy Advisory Committee (APAC). The first author received funding from Dietitians Australia to lead the development of this paper.

## AUTHOR CONTRIBUTIONS

All authors contributed to the design and approach taken to develop this position paper. LB drafted the manuscript and all authors contributed to revisions. All authors approved the final manuscript before submission. The content has not been published elsewhere. The authors acknowledge Dietitians Australia's Food and Environment Interest Group members for their advocacy efforts for the development of this position paper, and their input to the final manuscript.

## Supporting information


**Data S1**: Supporting Information

## Data Availability

The data that support the findings of this study are available from the corresponding author upon reasonable request.
