# Supplementary material for: Dietitians Australia position statement on healthy and sustainable diets
Source: Nutr Diet. 2022 Mar 1;79(1):6–27. doi: 10.1111/1747-0080.12726 (PMC9311218; doi:10.1111/1747-0080.12726)
Supplement: Supplementary file 1 — Data S1: Supporting Information [file NDI-79-6-s001.docx]

**Research Question 1: What are the characteristics of healthy and sustainable diets?**

*Table 1: Search strategy for Research Question 1*

| *Database* | *Google Scholar* | *PubMed* |
| --- | --- | --- |
| **Search Date** | 23/11/2021 | 23/11/2021 |
| **Date Range** | 1/1/2012* - 23/11/2021 | 1/1/2012* - 12/12/3000 |
| **Search Terms** | (definition OR defining) (health OR nutrition OR nutritious) (sustainable OR ecological OR eco-friendly) (food OR diet) | (health OR nutrition OR nutritious) (sustainable OR ecological OR eco-friendly) (food OR diet) |
| **Filters** | Date range, limited to peer-reviewed publications in English | Date range, limited to publications in English, only systematic literature reviews |
| **Number of results** | ~848,000 | 345 |

**2012 is when FAO published their landmark definition of healthy and sustainable diets*

*Figure 1: Flow diagram of publication screening for Research Question 1*

**PART 2**

**PART 1**

**PART 3**

Records identified through complementary sources:

**Reference lists of included studies**

(n = 6)

**Expert working group members**

(n = 7)

*> Grey literature (n = 1)*

*> Peer-reviewed literature (n = 6)*

Records identified through database searching:

**PubMed** (n = 345)

*Note search was restricted to Systematic Reviews*

Records identified through database searching:

**Google Scholar** (n ~ 848,000)

*Note only first n=100 (most relevant) items screened*

Identification

Full-text articles excluded (n = 11)

Exclusion reasons:

Ineligible study type (n=1)

Not contributing to a new definition/ concept (n=7)

Not specific to healthy and sustainable diets (n=3)

Full-text articles excluded (n = 6)

Exclusion reasons:

Not contributing to a new definition/ concept (n=4)

Ineligible study type (n=2)

X

X

X

X

Titles and introductory text screened

(n = 345)

Titles and introductory text screened

(n = 100)

Included

Eligibility

Full-text articles assessed for eligibility

(n = 12)

Full-text articles assessed for eligibility

(n = 10)

Studies deemed eligible (n = 4)

Studies included in synthesis (n = 18)

Studies deemed eligible (n = 13)

Studies deemed eligible (n = 1)

**Research Question 2: What approaches are being taken by researchers to measure health and environmental sustainability outcomes of population diets?**

*Table 2: Search strategy for Research Question 2*

| *Database* | *Google Scholar (https://www.scholar.google.com.au)* | *PubMed* |
| --- | --- | --- |
| **Search Date** | 23/11/2021 | 23/11/2021 |
| **Date Range** | 1/1/2019* - 23/11/2021 | 1/1/2019* - 12/12/3000 |
| **Search Terms** | (measure OR metric OR model OR impact) (health OR nutrition OR nutritious) (sustainable OR ecological OR eco-friendly) (food OR diet) | (health OR nutrition OR nutritious) (sustainable OR ecological OR eco-friendly) (food OR diet) (measure OR metric OR model OR impact) |
| **Filters** | Date range, limited to peer-reviewed publications in English | Date range, limited to publications in English, only systematic literature reviews |
| **Number of results** | ~135,000 | 110 |

**2019 is when the EAT-Lancet published their planetary health diet, a reference diet used to quantify the impact of healthy and sustainable population diets*

*Figure 2: Flow diagram of publication screening for Research Question 2*

**PART 2**

**PART 1**

**PART 3**

Records identified through complementary sources:

**Reference lists of included studies**

(n = 7)

**Expert working group members**

(n = 26)

*> Grey literature (n = 2)*

*> Peer-reviewed literature (n = 24)*

Records identified through database searching:

**PubMed** (n = 110)

*Note search was restricted to Systematic Reviews*

Records identified through database searching:

**Google Scholar** (n ~135,000)

*Note only first n=100 (most relevant) items screened*

Identification

Full-text articles excluded (n = 1)

Exclusion reasons:

Focused on consumer attitudes/responses rather than metrics (n=1)

Full-text articles excluded (n = 1)

Exclusion reasons:

Ineligible study type (n=1)

Titles and introductory text screened

(n = 110)

Titles and introductory text screened

(n = 100)

Included

Eligibility

Full-text articles assessed for eligibility

(n = 3)

Full-text articles assessed for eligibility

(n = 8)

Studies deemed eligible (n = 7)

Studies included in synthesis (n = 42)

Studies deemed eligible (n = 33)

Studies deemed eligible (n = 2)

**Research Question 3: What evidence-based policy options exist to facilitate the uptake of healthy and sustainable diets in Australia?**

*Table 3: Search strategy for Research Question 3*

| *Database* | *Google Scholar*  *(https://www.scholar.google.com.au)* | *PubMed* |
| --- | --- | --- |
| **Search Date** | 28/11/2021 | 23/11/2021 |
| **Date Range** | 1/1/2015* - 28/11/2021 | 1/1/2015* - 12/12/3000 |
| **Search Terms** | (health OR nutrition OR nutritious) (sustainable OR ecological OR eco-friendly) (food OR diet) (policy OR act OR strategy OR plan OR scheme OR intervention OR law OR legislation OR regulation OR guideline OR action) | (health OR nutrition OR nutritious) (sustainable OR ecological OR eco-friendly) (food OR diet) (policy OR act OR strategy OR plan OR scheme OR intervention OR law OR legislation OR regulation OR guideline OR action) |
| **Filters** | Date range, limited to peer-reviewed publications in English | Date range, limited to publications in English, only systematic literature reviews |
| **Number of results** | ~870 000 | 255 |

**2015 is when the Sustainable Development Goals were published, triggering policy action at the International, Regional, National and Local levels to address issues of poverty, inequality, climate change, environmental degradation, peace and justice.*

*Figure 3: Flow diagram of publication screening for Research Question 3*

**PART 2**

**PART 1**

**PART 3**

Records identified through complementary sources:

**Reference lists of included studies**

(n = 11)

**Expert working group members**

(n = 35)

*> Grey literature (n = 7)*

*> Peer-reviewed literature (n = 28)*

Records identified through database searching:

**PubMed** (n = 255)

*Note search was restricted to Systematic Reviews*

Records identified through database searching:

**Google Scholar** (n ~870,000)

*Note only first n=100 (most relevant) items screened*

Identification

Full-text articles excluded (n = 6)

Exclusion reasons:

Ineligible setting (n=2)

Ineligible intervention (n=2)

Ineligible subject (n=2)

X

X

Full-text articles excluded (n = 7)

Exclusion reasons:

Ineligible intervention (n=5)

Ineligible setting (n=2)

X

X

X

Titles and introductory text screened

(n = 255)

Titles and introductory text screened

(n = 100)

Included

Eligibility

Full-text articles assessed for eligibility

(n = 13)

Full-text articles assessed for eligibility

(n = 15)

Studies deemed eligible (n = 8)

Studies included in synthesis (n = 61)

Studies deemed eligible (n = 46)

Studies deemed eligible (n = 7)
